# Cine delayed hyper-enhancement CMR imaging without additional scan time

**DOI:** 10.1186/1532-429X-16-S1-P200

**Published:** 2014-01-16

**Authors:** Azza S Hassanein, Ayman M Khalifa, Brian P Shapiro, El-Sayed H Ibrahim

**Affiliations:** 1 Helwan University, Cairo, Egypt; 2 Mayo Clinic, Jacksonville, Florida, USA

## Background

A typical CMR exam includes cine, tagging, first-pass, and delayed-hyper-enhancement (DHE) imaging. The DHE image is usually acquired at a single timeframe. Nevertheless, obtaining cine DHE images would provide both viability and functional information in single set of images without misregistration problems for better image interpretation. However, repeated breath-hold scans are required to obtain cine DHE images, which increases scan-time with varying level of tissue enhancement with time. In this work, we propose a new technique for generating cine DHE images based on the acquired DHE image and motion field derived from the corresponding tagged images.

## Methods

A mid-diastolic short-axis DHE image and the corresponding set of tagged images were acquired on 3T-MRI scanner. The corresponding cine images were acquired for reference. Image analysis has two steps: 1) apply the Lucas-Kanade optical-flow algorithm to the tagged images to extract the motion field of the myocardium through the cardiac cycle; 2) apply the measured motion field to the known (acquired) DHE image to generate the unknown DHE images. The infarcted regions were identified in the DHE images using the full-width at half-maximum method. Radial thickening was measured from both cine and DHE images to evaluate the generated images. Infarction transmurality, circumferential strain, and radial thickening were measured from the DHE, tagged, and cine images, respectively, and the relationships between the generated parameters were studied using regression and correlation analyses.

## Results

**Figure 1:**
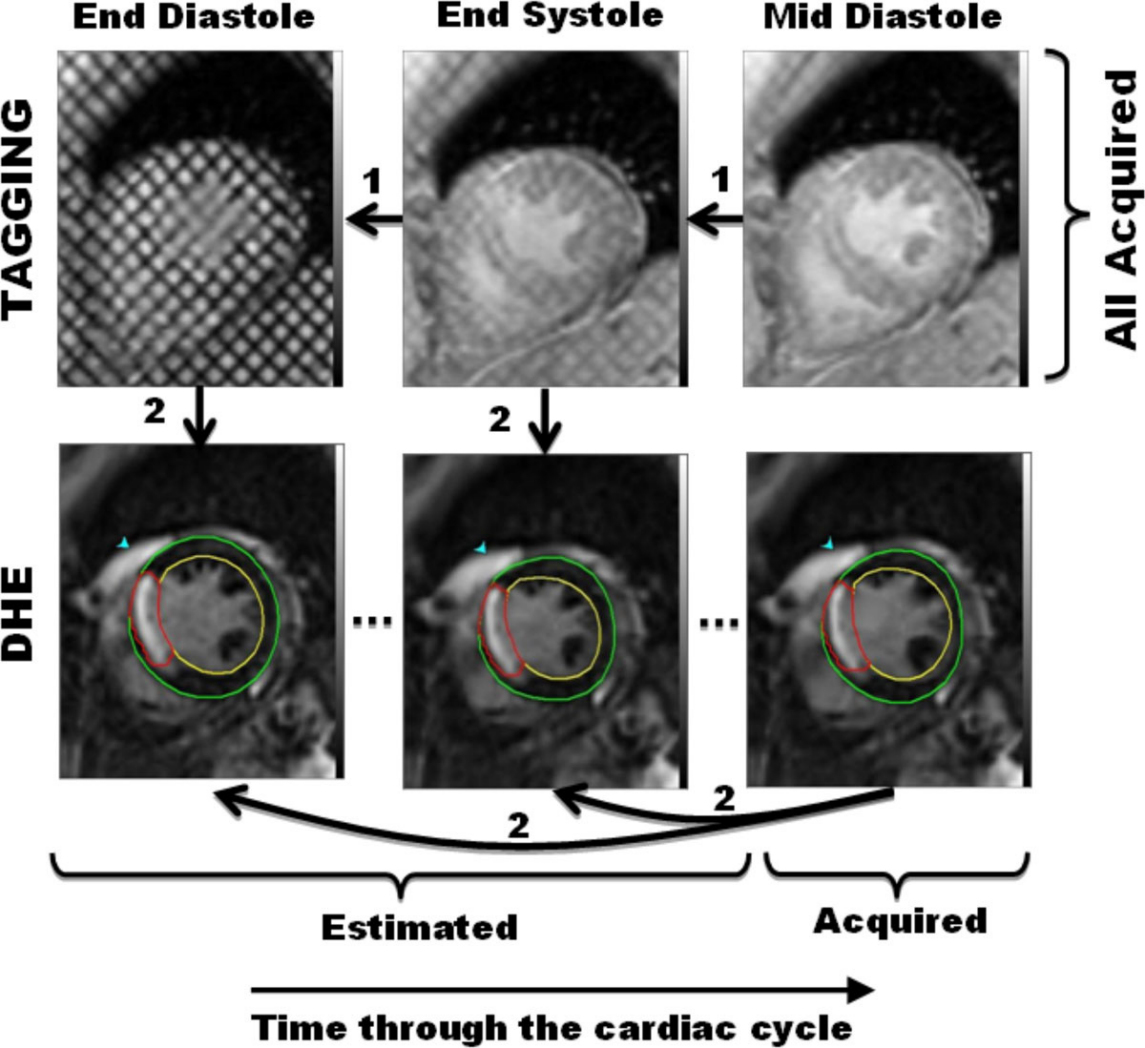
**Processing steps and results**. All tagged images (top) and one DHE image (bottom right) are acquired from the scan. Starting from the tagged image corresponding to the known DHE image (top right), optical flow is used to extract the motion field at all timeframes (step#1; arrows). Then, the measured motion fields are used to estimate the missing DHE images starting from the known DHE image (step#2; arrows). The endo- and epicardium are marked on the DHE images for measuring myocardial thickening. The infarction is identified using full-width at half maximum for measuring transmurality.

**Figure 2:**
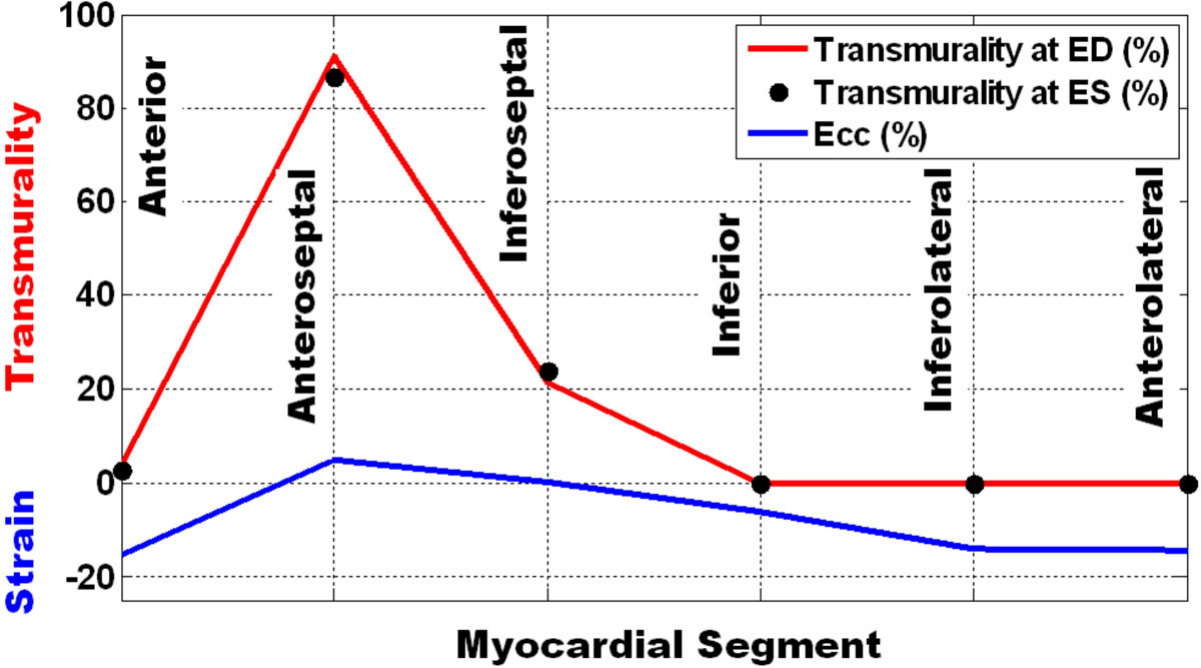
**Infarction transmurality and Strain**. The upper curve and black dots show excellent agreement between infarction transmurality calculated from the estimated DHE images at end-diastole and end-systole, respectively, for different myocardial segments. The lower curve shows circumferential strain with very good correlation with transmurality. Note that strain is represented in negative numbers, i.e. the lower strain, the better function.

Figure 1 shows tagged and DHE images. Figure 2 shows transmurality and strain at different segments. Peak infarction transmurality (circumferential strain) were 91% (0%) and 0% (-14%) in the infarcted and lateral regions, respectively. Myocardial thickening, calculated from the DHE images, showed good correlation with the results from the gold-standard cine images (r = 0.72). Further, there was an excellent agreement between ED and ES infarction transmurality calculated from the DHE images (r = 0.998). Infarction transmurality showed good correlation with strain (r = 0.81; y = 0.19x+11.27).

## Conclusions

The developed technique generates composite images that show both viability and functional information in one set of images, which is important for prognostic evaluation of the patient's condition, e.g. determining candidates for revascularization. The generated images could be also useful for differentiating between gadolinium recess in the muscle and myocardial scar, usually conducted by comparing cine and DHE images side-by-side. Future work includes further improvement of the optical-flow algorithm to minimize error propagation with time, and implementing the algorithm on large number of patients with different degrees of infarction.

## Funding

N/A.

